# RE-AIM in the Real World: Use of the RE-AIM Framework for Program Planning and Evaluation in Clinical and Community Settings

**DOI:** 10.3389/fpubh.2019.00345

**Published:** 2019-11-22

**Authors:** Bethany M. Kwan, Hannah L. McGinnes, Marcia G. Ory, Paul A. Estabrooks, Jeanette A. Waxmonsky, Russell E. Glasgow

**Affiliations:** ^1^Department of Family Medicine and the Adult and Child Consortium of Health Outcomes Research and Delivery Science, University of Colorado Anschutz Medical Campus, Aurora, CO, United States; ^2^Center for Population Health and Aging, Texas A&M University, College Station, TX, United States; ^3^Department of Health Promotion, College of Public Health, University of Nebraska Medical Center, Omaha, NE, United States

**Keywords:** RE-AIM, dissemination and implementation, program planning, evaluation, pragmatic

## Abstract

**Background:** The RE-AIM framework has been widely used in health research but it is unclear the extent to which this framework is also used for planning and evaluating health-related programs in clinical and community settings. Our objective was to evaluate how RE-AIM is used in the “real-world” and identify opportunities for improving use outside of research contexts.

**Methods:** We used purposive and snowball sampling to identify clinical and community health programs that used RE-AIM for planning and/or evaluation. Recruitment methods included surveys with email follow-up to funders, implementers, and RE-AIM working group members. We identified 17 programs and conducted structured in-depth interviews with key informants (*n* = 18). Across RE-AIM dimensions, respondents described motivations, uses, and measures; rated understandability and usefulness; discussed benefits and challenges, strategies to overcome challenges, and resources used. We used descriptive statistics for quantitative ratings, and content analysis for qualitative data.

**Results:** Program content areas included chronic disease management and prevention, healthy aging, mental health, or multiple, often behavioral health-related topics. During planning, most programs considered reach (*n* = 9), adoption (*n* = 11), and implementation (*n* = 12) while effectiveness (*n* = 7) and maintenance (*n* = 6) were considered less frequently. In contrast, most programs evaluated all RE-AIM dimensions, ranging from 13 programs assessing maintenance to 15 programs assessing implementation and effectiveness. On five-point scales, all RE-AIM dimensions were rated as easy to understand (Overall *M* = 4.7 ± 0.5), but obtaining data was rated as somewhat challenging (Overall *M* = 3.4 ± 0.9). Implementation was the most frequently used dimension to inform program design (*M* = 4.7 ± 0.6) relative to the other dimensions (3.0–3.9). All dimensions were considered similarly important for decision-making (average *M* = 4.1 ± 1.4), with the exception of maintenance (*M* = 3.4 ± 1.7). Qualitative corresponded to the quantitative findings in that RE-AIM was reported to be a practical, easy to understand, and well-established implementation science framework. Challenges included understanding differences among RE-AIM dimensions and data acquisition. Valuable resources included the RE-AIM website and collaborating with an expert.

**Discussion:** RE-AIM is an efficient framework for planning and evaluation of clinical and community-based projects. It provides structure to systematically evaluate health program impact. Programs found planning for and assessing maintenance difficult, providing opportunities for further refinement.

## Introduction

With the proliferation of evidence-based programs for improving population health, there is a greater concern with promoting the dissemination and implementation (D&I) of health programs (1). It is important to evaluate if these programs are being used, implemented as intended, and having the expected impact on health outcomes, to ensure we are investing in the best available programs and strategies (2). There have been numerous calls for more comprehensive use of dissemination and implementation science models, theories, and frameworks (3–5) to help understand how programs work, inform future interventions, and provide generalizable knowledge. To date, most assessments of the use of frameworks have been in research settings (6). This is also true of the RE-AIM framework, use of which in research grants and publications has been well-documented (7–9). There has been far less evaluation of use of RE-AIM in non-research settings. Such use is especially appropriate for RE-AIM, which is designed to be a pragmatic model (10).

We define “non-research” projects as programs and interventions intended for local quality or health improvement rather than generalizable knowledge (e.g., instituting evidence-based practices to improve patient outcomes vs. pursuing patterns of patient changes to advance a scientific question). Research may use random assignment to control conditions and be intended to establish efficacy or effectiveness. In contrast, non-research uses –which as discussed here, include quality improvement (QI), program or product evaluation, and demonstration projects. QI and program evaluations may have greater opportunity for adaptations and iterative refinement of protocols and intervention delivery. In both research and non-research contexts, the value of RE-AIM is in adding information on issues that are often not considered, such as outcomes involving representativeness and generalizability. Yet it is not known the extent to which and how RE-AIM is used for non-research purposes.

RE-AIM is a planning and evaluation model that addresses five dimensions of individual- and setting-level outcomes important to program impact and sustainability (11): Reach, Effectiveness, Adoption, Implementation, and Maintenance. Reach refers to the absolute number, proportion, and representativeness of individuals who participate in a given intervention or program. Effectiveness is the impact of an intervention on important outcomes and includes negative effects, quality of life, and economic outcomes. Adoption is the absolute number, proportion, and representativeness of settings and intervention agents who initiate a program. Implementation refers to the intervention agents' fidelity to and adaptations of an intervention and associated implementation strategies, including consistency of delivery as intended and the time and costs. Lastly, maintenance is the extent to which a program or policy becomes institutionalized or part of the routine organizational practices and policies. Within the RE-AIM framework, maintenance also applies at the individual level, and has been defined as the long-term effects of a program on outcomes after 6 or more months after intervention contact.

Systematic reviews of the research literature on RE-AIM have found that certain dimensions (effectiveness, implementation) are evaluated and reported more often than other dimensions (reach, adoption, maintenance)—with the primary underreporting in describing the representativeness of individuals and settings, maintenance of effects, costs, and sustained program implementation (7). To our knowledge, the only reported assessment of the use of RE-AIM in non-research settings has been in a series of articles coordinated by Ory et al. on use in aging and health programs (12). In an article on perceived utility of the RE-AIM framework (13), program implementers and administrators in 27 states were interviewed about use of RE-AIM as a guiding framework to plan, deliver and evaluate state-level delivery of a national evidence-based prevention initiative directed toward older adults. Findings suggested high perceived utility by key community stakeholders in using the RE-AIM framework in national initiatives for older adults. Although RE-AIM was viewed as a useful planning, implementation, and evaluation tool, uptake was not universal across all dimensions, and difficulty was reported in applying the framework as a whole. A major conclusion from this study was the need for more tailored resources and technical assistance, something that the National Working Group on RE-AIM Planning and Evaluation Framework (www.re-aim-org) has been addressing (14, 15). Additionally, this paper called for additional assessments of the use of the RE-AIM framework in other funding initiatives as part of a quality assurance process to understand roll-out of evidence-based programming.

To understand the application of RE-AIM in non-research settings across different content areas, populations and settings, we designed a study to assess experience (understandability and usefulness) and methods and resources used to apply all five RE-AIM dimensions for program planning, evaluation, decision-making and improvement in non-research settings.

## Materials and Methods

### Design

This is a cross-sectional, retrospective mixed-methods evaluation of use of RE-AIM for program planning and evaluation in clinical and community settings. We identified eligible programs and representatives using purposive and snowball sampling. We administered structured interviews to program representatives to assess both qualitative perceptions and quantitative ratings of the usability and usefulness of RE-AIM. Open-ended questions were used to elaborate upon and explain quantitative ratings, consistent with an explanatory concurrent mixed methods design. Study procedures were approved by the Colorado Multiple Institutional Review Board in June 2018.

### Participants and Eligibility

Our goal was to interview 15–20 representatives from projects or programs that used RE-AIM for planning and/or evaluation in clinical or community settings. Eligibility criteria for interviewees included a lead or supportive role in the local planning, implementation, and/or evaluation of a health-related program or intervention (e.g., program director) and self-reported use of RE-AIM.

### Sample Identification and Recruitment

It is challenging to review and evaluate non-research use of models and theories (13). There are no repositories or databases such as PubMed or federal grants to search, and publications in the academic literature or gray literature are rare. Therefore, pragmatic methods such as purposive and snowball sampling (asking those interviewed for recommendations of other potential participants) can be used. This makes it difficult, however, to establish a denominator or response rate.

We used multiple strategies to identify and recruit interviewees. First, we developed a brief survey sent to contacts at agencies that we had reason to believe had funded, conducted, or organized health-related programs designed primarily for local QI or community health impact (purposive sampling). Agencies included health foundations, health systems, state health departments, and national health agencies in the U.S and internationally. We targeted both agencies and individuals known to us (experienced RE-AIM scholars) to have required or encouraged use of RE-AIM for planning or evaluation of supported projects, as well initiatives in which use of RE-AIM was unknown. The identified contact persons from target agencies were sent an email with a survey link. Those that did not reply after 10 days were sent a reminder e-mail.

The survey asked, “Have any of your grantees ever actually used RE-AIM, in whole or in part, as a program planning or evaluation framework for one or more health-related programs or initiatives?” Respondents indicated how many programs or initiatives had used RE-AIM, and described projects that “Primarily aimed to improve the health or well-being of a specific community or population” and had used RE-AIM. With respondents' permission, we followed up via email to request introductions or contact information for representatives from relevant projects.

Names of representatives from relevant projects were also obtained through personal contacts, PubMed and Google Scholar searches, emails to the RE-AIM working group, and nomination by RE-AIM researchers and interviewees (snowball sampling). Literature search terms included “RE-AIM,” “health” and “evaluation”; we reviewed abstracts to identify potentially eligible projects, and emailed lead authors.

In total, we emailed 95 people with invitations to complete the survey (for agency representatives) and/or participate in the interview (for program representatives; some people represented both). Many were multiple potential contacts for the same program. Of the 35 surveys completed, 19 reported their grantees had used RE-AIM; 14 indicated they would provide introductions to project representatives. Of the 17 interviews conducted, 8 resulted from contacts identified by those who were sent the survey, 1 resulted from nominations from other interviewees, 2 resulted from emails to the RE-AIM working group, and 2 resulted from personal contacts. Four of the interviewees were co-authors on this paper, who are experienced evaluators and had used RE-AIM for eligible projects. All but one interview was conducted by a trained research assistant; one was conducted by the lead author.

### Outcomes and Data Collection Tools

Data were collected using a structured interview guide (Supplementary Material). Additionally, we requested copies of any public documents, project summaries, and reports involving the RE-AIM framework on their project. Participants reported if they had ever been involved in a “non-research” project that used RE-AIM, in whole or in part, as a program design or evaluation framework; if they had been involved in more than one such project, they were asked to consider the most recent project for the remainder of the interview. We audio recorded the interviews, and kept detailed notes. We appended our notes after the interview by reviewing the recordings.

#### Quantitative Outcomes

For each RE-AIM dimension, participants reported whether they used the dimension for initial planning or program design, evaluation, or both, and described how they measured or otherwise operationalized the dimension. Additional details about measurement/operationalization were gathered from written reports or documents provided by the interviewee. Interviewees rated usability and usefulness of the five RE-AIM dimensions using 5-point Likert-type scales: “Dimension was easy to understand” (1 very difficult – 5 very easy); “Getting the data to assess this dimension was easy” (1 very difficult – 5 very easy); “Did consideration of this dimension inform initial program design?” (1 not at all – 5 extremely); “Was consideration of this dimension important for decision making during the program?” (1 not at all – 5 extremely).

#### Qualitative Outcomes

Open-ended questions in the interview guide addressed the following topics:

- Description of the health program, project, or initiative, its intended audience and outcomes, and its origin and funding;
- Rationale, purpose and funding for use of RE-AIM;
- Experience with and methods used to apply the five RE-AIM dimensions;
- Receipt and nature of consultation, published guidance, online resources, training, and technical assistance; and
- Recommendations for improvement in the model itself and guidance materials.

### Analyses

#### Quantitative Analyses

For quantitative ratings, we calculated descriptive statistics including means, medians, standard deviations and ranges for 16 (of 17) interviews. One interview was excluded from the quantitative analysis as it covered use of RE-AIM for a series of projects, rather than a single project, and hence was not comparable for quantitative analyses.

#### Qualitative Analyses

For qualitative data, the research assistant who conducted the interviews organized her notes into a case-based matrix by topic (project health topic and setting, program and RE-AIM use funders, RE-AIM impact and usefulness, challenges, overcoming challenges, use of educational resources, and recommendations for improvement). Another team member coded the data within each topic area and identified themes. We used a qualitative descriptive approach to content analysis (16). All 17 interviews were included in the qualitative analysis.

## Results

### Description of the Sample and Use of RE-AIM

As shown in Table 1, the interviews represented a diverse group of projects and organizations. The interview participants were most often from universities (62%), especially schools of public health. Project locations where programs were conducted were primarily in community or public healthcare settings—and not associated with research funding. Programs were located in the U.S (Colorado, Maine, Georgia, Wyoming, Texas, Alaska, Nebraska, Washington State, national programs), and outside the U.S (Guadalajara, Amsterdam). Projects addressed several different content areas, and most (71%) focused on prevention or management of multiple health conditions or behaviors. Funding came from several sources, with the most frequent being U.S. public health funders such as the Centers for Disease Control and Prevention (CDC) or state health departments. It is notable that 6 programs had either had no funding or combined funding from multiple sources, and 2 were internally funded. Finally, these programs made modest use of RE-AIM resources, including www.re-aim.org (9 programs) or formal training or consultation (7 programs).

**Table 1:** Characteristics of organizations and projects interviewed.

**Organization type**	**No. of 16**
U.S. university	8
Government (non-research agency)	3
Foreign university	2
Research evaluation center	2
Non-profit organization	1
**Project content area**	
Multiple	6
Chronic disease or aging	5
Prevention	4
Mental health	1
**Funding source**	
CDC or state health	6
National health organization	4
Internal or none	3
Multiple	3
**RE-AIM resources used**	
www.re-aim.org	9
Consultation	7

Table 2 summarizes which RE-AIM dimensions were addressed in each program, for planning or evaluation. Respondents reported RE-AIM was used more comprehensively for evaluation than for planning. Organizational factors such as adoption and implementation were used most frequently for planning while individual factors such as reach and effectiveness were used less frequently. In terms of evaluation, all dimensions were reportedly used in most programs.

**Table 2:** Use of RE-AIM dimensions for planning and evaluation (no. of 16 possible).

**RE-AIM dimension**	**Planning**	**Evaluation**
Reach	9	14
Effectiveness	7	15
Adoption	11	14
Implementation	12	15
Maintenance	6	13

Tables 3, 4 summarize exemplar projects' application of RE-AIM in Clinical and Community Settings, respectively.

**Table 3:** Definitions and measures for RE-AIM applications in a clinical setting.

	**Clinical setting case study**
Project type	• Clinical project to ensure exercise is addressed in routine medical care
Why use RE-AIM?	• Used RE-AIM for evaluation purposes. • Used as part of a process evaluation. • Also used to shape interventions and projects.
Reach	• Defined as type of patients reached by the intervention, including characteristics of patients and how generalizable to the target population for the program. • Measures: Patient demographic information.
Effectiveness	• Defined as expected or perceived outcomes by clinicians. • Examined the difference between intervention effectiveness and implementation effectiveness. • Measures: Questionnaires and in-depth interviews with the clinicians.
Adoption	• Defined as what sorts of departments are willing to use the new intervention and what sorts of clinical/staff are willing to use it • Measures: Questionnaires and in-depth interviews with clinicians. Demographic data based on field notes, talking to people in recruitment.
Implementation	• Defined as clinician adherence to protocols for implementation and intervention. • Measures: Questionnaires, interviews, and observations.
Maintenance	• Defined as intended use after evaluation at the clinician level, including recommendations for others to use the intervention and anticipated benefits other settings. • Measures: Questionnaires and interviews.

**Table 4:** Definitions and measures for RE-AIM application in a community setting.

	**Community setting case study**
Project type	• Older Adults Community Living: Fall prevention and exercise programs
Why use RE-AIM?	• Used RE-AIM as a framework for evaluation. • Used RE-AIM to describe dissemination for a regional project.
Reach	• Defined as types of people who participated in the prevention programs, and assessment of who was more likely to participate in each of two exercise programs. • Also defined as extent to which programs were covering all counties in their service area, covering the hotspots or places within certain miles of a hospital or well-known health clinic, or whether areas were missing. • Used to help an organization when they were making a decision about what programs best suited their population, whether it was by age category, by income level, or education level. • Measures: Participant, Host site, and Program leader surveys (pretest posttest) including demographics, such as gender, race, income level of participants as well as who did not participate and who were missing, over 2 years. Geocoding using zip codes to evaluate the site and how many people they served.
Effectiveness	• Given a focus on disseminating evidence-based falls prevention programs, there was less concern about the effectiveness. • Defined as quality assurance throughout the project, attitudes toward perceived effects on falls efficacy at end of the program. • Fidelity check as part of quality assurance, making sure that the program is working as intended. • Measures: Quality assurance check-ins and survey measures on falls efficacy and falls risk factors and ensured that outcomes were matching up with national outcomes as a fidelity check.
Adoption	• At the organizational level, defined as characteristics of organizations and implementation sites, including those invited to participate and their organizations; and reasons for adoption or non-adoption. • At the participant level, defined as characteristics of those who participated, who was hesitant and reasons for hesitations. • Measures: Survey and anecdotal information; the collection of the host information form and the population that they served. Collected the zip code location of the sites to garner information about the population served, where they were located, if they received any government funding, and how each site was set up.
Implementation	• Defined as program attendance and the number of programs or classes offered, and having quality assurance measures (such as using a checklist) in place to ensure fidelity to program protocol. • Measures: Individual level surveys to measure program attendance and the number of programs or classes offered, using attendance logs and completion logs. Fidelity observations for new program leaders.
Maintenance	• At the individual level, maintenance defined as evidence on sustaining benefits and participants' intentions to continue the program. • At the site level, sustainability defined as evidence of organizations embedding these programs into their routine operations and budgets. • Measures: Questionnaires and interviews with organization representatives.

### Quantitative Ratings of RE-AIM Dimensions

Table 5 summarizes respondent ratings on the five RE-AIM dimensions for: perceptions of ease of use, ease of data acquisition, use in program design decisions, and importance for decision making. All domains were rated as easy to understand (4.3 or higher on the 5 point scale), with few differences across dimensions. Obtaining data was rated as moderate across dimensions (average of 3.4), but somewhat more difficult for maintenance. The largest difference across dimensions was on the rating of “informed program design,' on which Implementation was rated more highly than the other dimensions (4.7 of 5 vs. 3.0–3.9 for all other dimensions). All dimensions, except maintenance (3.4/5) were reported to be important for decision making (all >4/5).

**Table 5:** Ratings of RE-AIM dimensions on ease of understanding and getting data; consideration for design and decision making.

**RE-AIM dimension**	**Easy to understand**	**Ease of getting data**	**Considered in program design**	**Important for decision making**
	**M (SD)**	**M (SD)**	**M (SD)**	**M (SD)**
Reach	4.3 (0.8)	3.5 (1.1)	3.5 (1.6)	4.0 (1.4)
Effectiveness	4.7 (0.6)	3.4 (1.3)	3.9 (1.6)	4.1 (1.4)
Adoption	4.2 (0.9)	3.7 (1.2)	3.9 (1.3)	4.1 (1.3)
Implementation	4.7 (0.5)	3.4 (1.1)	4.7 (0.6)	4.2 (1.3)
Maintenance	4.4 (0.8)	3.1 (1.1)	3.0 (1.4)	3.4 (1.7)
Overall	4.7 (0.5)	3.4 (0.9)	3.6 (1.6)	3.6 (0.8)

*Each item measured on a scale of 1 (“very difficult” or “not at all”) to 5 (“very easy” or “extremely”) scale*.

### Qualitative Themes

We identified themes related to the usefulness of RE-AIM for real-world projects, including its impact on planning and usefulness for evaluation and program refinements over time, challenges in using RE-AIM and strategies for overcoming those challenges, resources used for learning about and applying RE-AIM, recommendations for improvement, and how use of RE-AIM is funded.

#### RE-AIM Impact and Usefulness

RE-AIM was reported to be a useful organizing framework or “roadmap” for planning (impact and usefulness theme 1). An interviewee explained, “[RE-AIM] provides a nice roadmap with all the components of program development that you need to consider.” RE-AIM helps to ensure consideration of adoption, implementation and sustainability, reaching the right audiences, and ensuring you have been clear about who will benefit and expected outcomes, as well as potential unintended consequences (e.g., attending to reach and potential health disparities). An interviewee described the value of the “reach” dimension for attending to health equity, “It was a moral imperative to check in on what we were doing…. We wanted to use this to inform, how can we reach those vulnerable populations, how can we reach rural populations, how can we reach those disadvantaged or untouched sectors of the community.”

RE-AIM was reported to help with focusing on context and setting, and implications for what works in “real life” (impact and usefulness theme 2). Specifically, understanding who is or isn't adopting or delivering the program well, what's required for sustainability, and making refinements to the program over time to ensure overall quality. Finally, RE-AIM was described as a practical, familiar, easy to understand and well-established D&I framework (impact and usefulness theme 3). One interviewee indicated, “if you are wanting a framework or conceptual model, this is a good one” since many people are familiar with it and it's fairly straightforward, and helpful for coordinating and communicating with program implementers. Furthermore, “I'm from the public health field and it's always nice to have a framework, theory or model on which you base your decisions on. So that was a big value-add for me was to have an established D&I framework that was known in the field and highly applicable to our project.”

#### RE-AIM Challenges

Interviewees reported challenges with understanding the differences among the RE-AIM components (challenges theme 1). Notably, respondents reported that there are “fuzzy boundaries” between adoption and implementation, adoption and reach, and reach and effectiveness (e.g., which dimension it is when the outcome is number served). A respondent noted, “I just think that distinguishing between when does somebody move from being an adopter to an implementer, I find that a fuzzy boundary.” There can be lack of clarity on the unit of analysis (e.g., participant vs. system/organization), as well as defining terms and figuring out relevance to a specific program. For example, in a program focused on recruiting staff members to deliver a program to parents, the staff participation would be the measure of *adoption* and parents contacted the measure of *reach*. Second, there can be difficulty with data acquisition, as in a source of data exists but access was slow or limited (challenges theme 2). Specifically, it was especially hard to get electronic health records (EHR) data that allowed linking patients over time to track clinical outcomes longitudinally. One person summarized the problem as, “Electronic health records are not set up to extract the data for these [RE-AIM] measures.” As in research and evaluation in general, interviewees struggled to schedule interviews with busy staff and providers, and often found participants did not want to spend a lot of time answering questions. A parallel data challenge theme concerns when a source of data doesn't exist (challenge theme 3); this includes getting denominator data to estimate reach and adoption, knowing who does not get into a program, getting demographic data for characterizing sites and participants, and measuring maintenance. For example, “Often there is no denominator known, so you want to describe our reach but don't know your full population so you cannot say if you did well on reach.” Finally, interviewees reported practical and logistical issues with RE-AIM evaluations—likely not specific to RE-AIM itself, but to the nature of evaluation (challenge theme 4). These issues included few resources, changing organizational priorities, staff turnover, and frustration that it can take a long time to see impact at the patient level.

#### Overcoming Challenges

To overcome challenges, interviewees reported that being flexible and adapting the approach over time to best fit your needs and purpose was helpful (overcoming challenges theme 1) but also resulted in modifications to RE-AIM definitions. One participant described this as “the way we're interpreting it (adoption) is probably not pure RE-AIM so we might be taking some liberties because … you're usually looking at adoption as sort of the analog to reach, what sort of percentage of all the eligible health systems that you approached actually signed on to do it. Respondents noted the liberal use of RE-AIM while focusing on one's purpose: “The way we used some of the reach data as a metric for taking a look at fidelity and seeing if, for example, one clinic is really falling short.” Some reported using a trial and error approach to methods and measures, being open to changing methods over time, and going back and forth with sites to figure out what will work from a measurement perspective. For instance, “In terms of maintenance … we had to expand beyond the cancer center to include all hospital inpatients.” In addition, interviewees suggested putting in the effort for careful planning up front (overcoming challenges theme 2). Specifically, spending time at the beginning to define your terms, develop relationships with sites and participants, and figure out what's realistic at the outset for both evaluation and for the program (e.g., what are realistic numbers for reach?). For instance, “Getting the data all depends on the previous steps. How you use it, how you define it, and what methods. It's worth spending a lot of time on designing that part so it's easy to collect.” Finally, interviewees advised learning more about the RE-AIM dimensions and how they have been applied in other real-world projects (overcoming challenges theme 3). This can help clarify definitions—such as one interviewee who reported that reading examples helped to clarify that adoption refers to the provider or setting level or that maintenance can refer to the organization level as well as the individual level.

#### Educational Resources

Interviewees reflected upon their use of the website, trainings or formal education, consultation or technical assistance, and publications to learn about and guide application of RE-AIM. Most interviewees were familiar with the website and had used or visited it in the past (14). They found it useful for finding definitions of terms and finding publications on RE-AIM (e.g., for examples and materials used in previous projects). Sources of training and formal education included graduate coursework (e.g., public health programs, D&I courses); lectures, conference presentations and webinars; and workshops from RE-AIM experts. They recommend promoting existing resources like recorded webinars so that others can benefit from them. Consultation and technical assistance included brief discussions to in-depth collaboration with RE-AIM experts, having RE-AIM experts walk them through the website, as well as peer-to-peer and internal organizational expertise in RE-AIM. Publications found to be most useful to guide understanding and applying RE-AIM in non-research settings included the original 1999 paper (11), the pragmatic applications in clinical and community settings paper (10), RE-AIM systematic review (7), RE-AIM for environmental change and health paper (17), the practical, robust implementation and sustainability model (PRISM) paper (18), and the use of RE-AIM for chronic illness management research paper (19).

#### Recommendations

To help promote ease of use of RE-AIM for community and clinical programs, interviewees had several recommendations for RE-AIM developers. First, highlight real-world projects on the RE-AIM website that do a good job linking measures to the constructs and giving concrete examples. Describe tools and measures that can be adapted—especially for measuring and distinguishing reach (especially denominators), adoption and maintenance. For instance, it is helpful “To have some good examples of tools people use to capture some of the dimensions especially those that are a little bit harder to capture like the maintenance piece for institutions or the adoption aspect.” Emphasize that RE-AIM can be used pragmatically by only assessing constructs that are a priority for your stakeholders and/or aligning RE-AIM constructs with metrics stakeholders care about. This allows practice settings to plan strategies that can address each RE-AIM dimension without feeling obligated to collect data across all dimensions. Users of the framework can benefit from clarifying when RE-AIM (vs. another framework) may be most applicable. For example, “I think now that there are so many frameworks out there I think it's just helpful to understand…how is RE-AIM different, when is it appropriate to use RE-AIM, when is appropriate to use other frameworks…. It's still pretty driven toward the research community, but it can be used outside of the research community.” Interviewees asked for better placing RE-AIM in context by explaining how RE-AIM factors influence and relate to each other, and formally integrating contextual factors and consideration of facilitators and barriers to adoption, implementation and maintenance into RE-AIM.

#### Funding RE-AIM in Community and Clinical Settings

Finally, interviewees described funding sources for applying RE-AIM, which sometimes differed from the funding for the program itself. Sources included federal grants, national agencies, internal health system funds (especially those at academic health centers and integrated health systems), foundations, state health departments, and commercial companies. The type or degree of support included small allocations from internal funds or carved out from larger grants, seed grants and case studies specifically geared toward evaluation, “leftover money” at the end of the year, or work done as part of regular employment or job duties.

## Discussion

Although challenging to identify programs using RE-AIM in non-research settings, our multiple recruitment approaches identified 17 non-research programs in “real world” clinical and community settings that had used RE-AIM for planning and/or evaluation. These programs included funders as well as universities, and government and community organizations. Our mixed methods assessment of RE-AIM use revealed that most RE-AIM dimensions have been used and found to be useful for planning and evaluation across diverse content areas, programs and settings. Qualitative findings show that RE-AIM is a well-known and easy to explain organizing framework or “roadmap” for planning, especially with regard to encouraging consideration of context and setting, and implications for what works in “real life.” These results are complementary to the more quantitative reviews that have been conducted on the formal research literature on use of RE-AIM. For instance, Vinson et al. found it to be one of the most frequently used implementation science frameworks in research grant proposals (20).

However, in contrast to the literature on research using RE-AIM, the program members interviewed for this report tended to use all RE-AIM dimensions for evaluation (7, 9, 21). In particular, these non-research users used Adoption and Maintenance dimensions in 100% and 75% of their applications, respectively, rates higher than reporting on these dimensions in the research literature. Key informants indicated that they used fewer of the dimensions during the planning period than the evaluation period. Of note, effectiveness and maintenance were considered in fewer than half of the program planning processes. It may be that when selecting programs for implementation the stakeholders began planning for implementation of evidence-based interventions—and as such felt that planning for adoption and implementation factors were the most important to address in planning to ensure the evidence-based approach would achieve the same magnitude of effect.

Some of the most frequent challenges to applying RE-AIM identified in these real-world applications were the same as those identified in the research literature, namely difficulty distinguishing Reach from Adoption, and specifying denominators for measures of Reach (10, 22). Despite consistent endorsement of RE-AIM as easy to understand or explain to program implementers, the nuances among dimensions can be—as one interviewee said—“tricky” to distinguish in practice. Further, despite the high use of all dimensions in evaluation across programs, there were challenges in proactive data collection—both from existing sources and gathered directly from participants. This suggests that even when using RE-AIM for planning, operationally evaluating each dimension can be complex.

It is informative to compare our results to those of Ory et al. (13) who evaluated use of RE-AIM for a larger number of settings (*n* = 27 states, with multiple delivery channels in each state) that were all part of one large national project to enhance physical activity. Similar to that report, we found that most RE-AIM dimensions were rated as relatively easy to understand and that RE-AIM was used consistently for both planning and evaluation. This stands in contrast to the research literature, in which RE-AIM has been used much more often for evaluation, despite documented successes in applying it for planning (23). The Ory et al. evaluation focused more on general experience applying RE-AIM using the framework as a whole, while our study delves into more specific evaluation of the use and helpfulness of the various RE-AIM dimensions in variety of different program areas (13).

We identified a number of themes related to challenges in using RE-AIM and strategies for overcoming those challenges in real-world settings. Most of the challenges reported are not unique to planning and evaluation of “real-world” programs—even research projects struggle with understanding RE-AIM dimensions, acquiring high-quality, longitudinal data, and maintaining commitment from staff and leadership. Similarly, as with any program evaluation, program planners and evaluators should engage stakeholders early in the process to ensure mutual agreement on defining outcomes of interest and establishing feasible measures and data sources for those outcomes. Strategies for overcoming challenges specifically aligned with using RE-AIM for non-research projects include being flexible and adapting the RE-AIM measures and priorities over time to best fit local and emergent needs and purpose. Program evaluation has more flexibility in this regard than does research.

Overall, RE-AIM appears to be applicable in non-research settings and to be helpful for pragmatic use (10) in projects that do not have large evaluation budgets. Respondents to our interview made moderate use of various resources on RE-AIM, including www.re-aim.org, but felt that more specific training in its use and case examples of how it has been applied in other non-research projects would be beneficial.

One notable recommendation for RE-AIM developers was to explicitly integrate RE-AIM with factors related to context and setting. This in fact has been done—the Pragmatic, Robust Implementation and Sustainability Model (PRISM) is an emerging D&I science framework that focuses on multiple factors related to context and setting (18, 24) that impact RE-AIM outcomes, but this expansion is not widely known. Such factors include the external environment, organizational and patient/recipient characteristics, and the implementation and sustainability infrastructure. For example, Liles et al. found that use of PRISM facilitated adoption of a new colorectal cancer screening intervention (24). In general, the PRISM framework focuses on contextual factors related to RE-AIM outcomes. While maintenance (at the setting level) in RE-AIM is defined as continuation after 6 months or longer following completion of funding support, newer conceptualizations of sustainability and the “implementation and sustainability infrastructure” component of PRISM focus on longer term sustainability.

### Limitations and Next Steps

This study has limitations including lack of a tightly defined sampling due to lack of searchable databases of non-research applications of RE-AIM (or other D&I frameworks). Although our records show we attempted to contact 95 people, which led to 17 interviews (16 of which reflected distinct programs), it was not possible to calculate a true response rate. There is no clear denominator for those eligible to participate. While this study includes a relatively small sample of informants, the interviews spanned 16 distinct programs and initiatives across a variety of health domains and clinical contexts. As is typical of qualitative research, we focused more on depth of understanding experiences with RE-AIM rather than breadth (25). Strengths of the study include its focus on pragmatic, non-research application and the mixed methods assessment. Unfortunately, the small number of interviewees precluded comparisons on RE-AIM use among programs with different types of funding or from different geographic locations. Future research is recommended to replicate and extend these findings at a future time to assess longitudinal trends, and development and evaluation of more specific training strategies for applying RE-AIM.

## Conclusions

RE-AIM can be a useful organizing framework or “roadmap” for planning and evaluation of implementation of health programs in clinical and community settings, especially given it helps focus on contextual and setting factors that have implications for what works in “real life.” As a practical, familiar, and easy to understand D&I framework, it is a good choice for a planning or evaluation framework in real-world settings. RE-AIM is generally not seen as an alternative to more traditional program evaluation methods, focusing on effectiveness, but as a way to broaden and contextualize results, with a focus on population impact (15). Projects using RE-AIM are not immune to the usual challenges of planning and evaluation, including data acquisition and availability, lack of resources, and changing priorities and staffing over time. It can also be difficult to understand the nuances and distinctions among the 5 RE-AIM dimensions, and figure out how each dimensions applies and can be measured in a given context.

To address these challenges, those planning to use RE-AIM may benefit from reading key papers on pragmatic application of RE-AIM (8–10) and not just the original 1999 paper; reviewing examples on the RE-AIM.org website; and seeking out consultations from experts. Then, be realistic about what can be done to measure RE-AIM dimensions, and be flexible to change over the course of the project in how RE-AIM is used. Finally, RE-AIM can serve as a tool for organizing and informing decision making before, during, and after program implementation. Specifically, funders can include RE-AIM as an expectation in grant applications to help systematically collect information on health program impact across multiple grantees (26). Additionally grantee organizations or evaluators can use RE-AIM as a tool for understanding what is working/not working within their programs and use this information to plan for quality improvement activities as well as long-term program sustainability.

## Data Availability Statement

The datasets for this study can be requested from the corresponding author with appropriate approvals.

## Ethics Statement

The studies involving human participants were reviewed and approved by the Colorado Multiple Institutional Review Board. Written informed consent for participation was not required for this study in accordance with the national legislation and the institutional requirements.

## Author Contributions

BK, RG, MO, PE, and JW contributed to the conceptualization of the project, study design and development of data collection materials, and recruited participants. BK and HM prepared IRB documents and collected data. BK, HM, and RG analyzed the data. BK and RG drafted the manuscript, and all authors edited and approved the final draft.

### Conflict of Interest

The authors declare that the research was conducted in the absence of any commercial or financial relationships that could be construed as a potential conflict of interest.
