# Supplementary material for: RE-AIM in the Real World: Use of the RE-AIM Framework for Program Planning and Evaluation in Clinical and Community Settings
Source: Front Public Health. 2019 Nov 22;7:345. doi: 10.3389/fpubh.2019.00345 (PMC6883916; doi:10.3389/fpubh.2019.00345)
Supplement: Supplementary file 1 [file Data_Sheet_1.docx]

**Usability and Usefulness of RE-AIM for Non-Research Projects**

This interview is about your experience with use of the RE-AIM framework (Reach, Effectiveness, Adoption, Implementation, and Maintenance) for program and policy design and evaluation. We are interested in hearing about projects *outside of research contexts* – that is, projects in which the primary goal is impact on local health rather than creating generalizable knowledge. Note that having published does not necessarily make your project “generalizable knowledge”. We plan to use this information to help guide analysis of how health-related programs and policies in non-research settings use RE-AIM, and how guidance on the application of RE-AIM outside of the research context could be improved. For this interview, we distinguish between the use of RE-AIM for program planning, program evaluation, or both planning and evaluation. This interview should take no more than 60 minutes. We may use this information in a published paper. We will not identify you or your project by name or other distinguishing characteristics without your permission. So that we may ensure we have captured your responses accurately, we would like to record this conversation. We will transcribe this recording and combine your information with others for analysis and reporting. Do you have any questions before we begin? Do I have your permission to record this interview?

1. First, tell us about yourself – what type of organization do you work for, what is your role there? How long have you been in that role? Has your role changed? What types of projects do you work on?
2. Have you ever been involved in a project that used RE-AIM, in whole or in part, as a program design or evaluation framework? How many projects come to mind? Please briefly list them.
3. Think about the *most recent* project in which you used RE-AIM for program planning or evaluation. Please tell me a bit about this project. *Probes:* health issue *- target behaviors or health problems meant to address.*, origin of the idea *- based on community needs assessment, funding announcement*, who implemented the project, role in the project
4. How was the project funded? Was there funding for the activities involving use of RE-AIM? Please explain.
5. Now, let’s talk about how RE-AIM was used in this project. *Probe: Pros and cons of the model, ease of understanding and applying the dimensions, origin of use of RE-AIM, previous experience with RE-AIM*
   1. First, please tell me, why did you decide to use RE-AIM for this project? What were the goals or objectives of using RE-AIM?
6. How did you apply RE-AIM? (Ask in conjunction with completing the table below)
   1. (Reach through Maintenance in table) Let’s go through each dimension of RE-AIM. Did the concept of [DIMENSION] inform program design or planning? Did you use [DIMENSION] for evaluation – such as a process or outcome measure? How did you measure [DIMENSION] – for instance, was it qualitative or quantitative? Upon measuring [DIMENSION], did you change the program to try to improve [DIMENSION]? (*Instruction to interviewer: ask questions for each dimension not used, document answers in table below; for any information already provided – either through materials provided in advance or in the interview so far, populate the table and confirm with respondent)* Next, please rate the usability and usefulness of each RE-AIM dimension on a scale from 1 (not at all useful/usable) to 5 (very useful/usable). (Usability – was it easy to understand and apply to your project; usefulness – did it improve your project or the impact of your project)
   2. (Now, for those dimensions you did not use: did you consider using it or not? If not, why not? If so, why did you decide against it? For example, did it not seem applicable to your project, or did it seem difficult to measure? (*Instruction to interviewer: ask questions for each dimension not used, document answers in table below; for any information already provided - either through materials provided in advance or in the interview so far, populate the table and confirm with respondent)*
   3. (Last row of table) Overall, on a scale from 1 (very difficult) to 5 (very easy), how easy was it to apply RE-AIM? What challenges did you experience? How did you address those challenges?
   4. Overall, on a scale from 1 (not at all useful) to 5 (very useful), how useful was RE-AIM? What effect did RE-AIM have on the impact of the project on the target health behaviors or health problems?

|  | How used  (Probe: **Initial planning/design or evaluation?** Did you change the program to improve dimension?) | Methods used  (How did you measure?) | Dimension was easy to understand  (1 very difficult – 5 very easy) | Getting the data to assess this dimension was easy  (1 very difficult – 5 very easy) | Did consideration of this dimension inform initial program design?  (1 not at all – 5 extremely) | Was consideration of this dimension important for decision making during the program?  (1 not at all – 5 extremely) |
| --- | --- | --- | --- | --- | --- | --- |
| - 1. Reach (who participated?) |  |  |  |  |  |  |
| - 1. Effectiveness (what outcomes?)   Quality of life,  Negative outcomes |  |  |  |  |  |  |
| - 1. Adoption (what settings **and** staff) |  |  |  |  |  |  |
| - 1. Implementation (what processes?)   Cost/time/burden  Did you customize framework to better fit project? |  |  |  |  |  |  |
| - 1. Maintenance (will program continue) |  |  |  |  |  |  |
| - 1. RE-AIM overall |  |  |  |  |  |  |

** send this table to interviewees in advance, explain this is to give some indication of what we’ll be discussing, we will go through this table together when we meet; they don’t need to complete this in advance but it may be helpful to take some notes and have this on hand for our conversation

1. Finally, please tell us what resources were used to guide application of RE-AIM in this project. This might include informational or reference materials, or consultation with an expert, or other resources.
   1. Was RE-AIM website, re-aim.org, used to guide application of RE-AIM? What could make the RE-AIM website more useful or easy to use? (E.g., short videos, interactive self-rating quiz, etc)
   2. Did the project team receive training on use of RE-AIM? If so, please explain.
   3. Did the project have a consultation or technical assistance from a RE-AIM expert? If so, with whom? Please explain. Marcia walked us through the website. Had a meeting, went through this exercise, imagined doing this in their communities – planning exercise, went through most of the dimensions, did that thinking about who would participate, what would be their issues or barriers, who would complete the program – big problem when people start the program vs who finishes – when you called it maintenance, are you talking about the participants themselves or the regional offices continuing the program – were often confused about that.
   4. Did you use other resources, such as *websites, textbooks,* other online or print materials? Manual or guide that talked about RE-AIM for the other project.

| (*Interviewer – list each resource used below, and then ask about usefulness for each)* | How useful was this resource, on a scale from 1 (not at all useful) to 5 (extremely useful)? |
| --- | --- |
|  |  |
|  |  |
|  |  |
|  |  |

1. Finally, do you have recommendations for ways that we could make RE-AIM more accessible, efficient, and useful for community or clinic-based programs, policies, or projects?
2. Anyone else we should speak with? For instance, has there been somebody else who has used RE-AIM for other projects in your setting that you think might be willing to speak with us? If so, would you make an introduction, please?
3. Anything I missed?

Thank you so much for your time today! We really appreciate your input.
